# Toothache—Inflammation of the Antrum

**Published:** 1864-06

**Authors:** Henry S. Chase


### TOOTHACHE—INFLAMMATION OF THE ANTRUM.

### A CASE.

BY HENRY S. CHASE, M. D.

Mr. F. called to have the right upper second molar extracted. He said the pain commenced three days ago, becoming gradually more severe, until he could "stand it no longer." Expecting to find a *decayed* tooth, what was my surprise to see that it was perfectly *sound*, as was also all the other teeth on that side of the mouth; neither was there inflammation of the gums. This tooth, and also the first molar, was slightly sensitive on percussion. Seeing that my patient had a severe nasal catarrh, I immediately made up my mind that the antrum was affected with the same disease, and had produced the dental irritation of which he complained. It is well known that the roots of the second bicuspid, first and second molars, often penetrate the antrum and participate in its diseases. I refused, of course, to extract the tooth, and after giving my patient an explanation, prescribed as follows: Take immediately and once an hour afterwards, one grain Mercurius Virus, 3d Dec. press., until better.

Twenty-four hours after my patient called, and with a smile said that his tooth ceased aching after the third dose, and that it was now perfectly well; also that his catarrh was better, and he wished a little more of the medicine to "finish up the cold."

*Independence, Iowa, April 8th,* 1864.

